# Clinical Efficacy and Biomechanical Behavior of Different Compression Systems in Venous Ulcers: Pressure, Stiffness and Healing

**DOI:** 10.3390/life16040585

**Published:** 2026-04-01

**Authors:** Juan Francisco Jiménez García, Maria Piedad García Ruiz, Francisco González Jiménez, Maria Gutierrez García, Jose Luis Jiménez Laínez, Mercedes Muñoz Condez, Ana Belen Fernández Ramirez, Francisco Pedro García Fernández

**Affiliations:** 1 Nursing Area, Almería Health District, 04009 Almería, Spain; juanfranjime@gmail.com; 2 Nursing Area, North Jaen Health Management Area, 23700 Jaen, Spain; 3 Nursing Area, Granada Metropolitan Health District, 18013 Granada, Spain; pacomollimolli@gmail.com (F.G.J.); epana0423@gmail.com (A.B.F.R.); 4 Nursing Area, Serranía de Málaga Health Management Area, 29400 Málaga, Spain; maguga052@gmail.com; 5 Nursing Area, Jaén-Jaén South Health District, 23009 Jaén, Spain; jimenezlainezjose@gmail.com; 6 Nursing Area, Málaga Axarquía Este Health Management Area, 29018 Málaga, Spain; mercemconde@gmail.com; 7 Departament of Nursing, University of Jaén, 23071 Jaén, Spain; fpgarcia@ujaen.es

**Keywords:** venous leg ulcers, multicomponent compression therapy, interface pressure, bandage static stiffness, edema reduction, wound healing, area reduction, advanced practice nurse, complex chronic wounds

## Abstract

Introduction: Venous leg ulcers are the most severe manifestation of chronic venous insufficiency, and their treatment is based on compression therapy, whose effectiveness depends on the magnitude of the pressure and the biomechanical properties of the system. Doubts persist about the actual correlation between interface pressure, bandage stiffness and clinical outcomes in real-world practice. Objective: To compare the clinical efficacy and biomechanical behavior of different multicomponent compression systems in venous leg ulcers, analyzing the relationship between interface pressure, static stiffness, edema reduction and variation in the wound area. Methodology: This is a prospective, observational and multicenter study in six districts/health areas of Andalusia, in adults with active venous ulcers attended by Advanced Practice Nurses in Complex Chronic Wounds. Several multi-component compression systems were applied, and interface pressure was monitored using Tight Alright^®^ at three points on the leg for 96 h, recording final pressure, static stiffness, perimeters and ulcer area. Results: All systems achieved a reduction in leg circumference, more marked at the proximal points, evidencing an overall decongestant effect. The correlation between final pressure and edema reduction was weak, and relevant differences were observed in the reduction in ulcer area, with Urgo K2 and CPK Compress 2 standing out with decreases of more than 50% compared to medium or low yields of other systems with comparable pressures. The static stiffness analysis showed specific patterns according to system and leg size, as well as a heterogeneous longitudinal distribution of pressure. Conclusions: The efficacy of compression in venous ulcers depends on both the interface pressure and the design and biomechanical behavior of the system, with clinically relevant differences between multicomponent dressings. Multipoint pressure and stiffness monitoring provides useful information to optimize system selection and support decisions based on biomechanical parameters and standardized clinical outcomes.

## 1. Introduction

Venous leg ulcer (VLU), the most severe manifestation of chronic venous insufficiency (CVI), is a significant public health problem, affecting approximately 1% of the adult population in developed countries [1,2,3]. The high frequency of venous disorders makes venous ulcers a problem of great health relevance, since they tend to persist over time, significantly affecting quality of life and representing a high social and economic cost. In addition, their tendency to recur is high, with recurrence rates that can reach between 40% and 70% [1,2,3,4]. Given this high clinical and social burden, it is essential to implement effective and sustained therapeutic interventions over time. In this context, compression therapy (CT) is universally recognized as the cornerstone in the prevention and treatment of VLU and CVI [4,5,6,7,8,9].

When the venous valve system and calf muscle pump malfunction, blood pools in the extremities, raising pressure in the venous system. This elevated pressure is transmitted to the dermal microcirculation, resulting in capillary damage and an increase in the leakage of fluid and macromolecules, such as fibrinogen, into the interstitial space. This leads to edema, inflammation, and ultimately ulcer formation. The accumulation of leukocytes in the lower limbs when they are in a dependent position, associated with venous hypertension, also contributes to trophic changes and skin damage through capillary occlusion and the release of inflammatory mediators [10].

By applying external pressure, compression therapy promotes the reduction in venous caliber, which limits reflux, increases the speed of blood return, and optimizes the function of the calf muscle pump, while helping to reduce peripheral edema and promote the healing process [6,7,8,11].

To ensure the effectiveness of compression therapy, it is necessary that the pressure, which is the essential element of the treatment, is distributed in a graduated manner, so that it remains between 40 and 45 mmHg at the ankle and progressively decreases towards the knee, which means that, when uniform tension is applied, the decreasing contour of the leg itself naturally generates the required pressure gradient [10,12,13].

Based on these physiological foundations, it is essential to consider that the clinical efficacy of compression does not depend only on reaching an adequate gradient, but also on the biomechanical properties of the system used. In this respect, the interface pressure and the rigidity of the bandage system are key parameters. Stiffness is usually assessed using the static stiffness index and dynamic stiffness index [11,13,14,15].

The evaluation and control of these parameters in clinical practice and research continue to be a considerable challenge. The proper application of bandages requires technical skill and continuous training, since the pressure exerted can vary between professionals and often does not reach the desired therapeutic values without the help of measuring instruments [8,12].

In addition, some of the available measuring devices, such as PicoPress^®^ or Kikuhime^®^, although widely used, show differences in accuracy and reliability [16,17]. In fact, pneumatic sensors can overestimate pressure, especially in areas of curvature, due to their thickness, complicating the comparison between studies [12]. For this reason, expert consensus highlights the need for thinner, more flexible sensors with multi-point measurement capability to obtain more accurate in vivo recordings [18].

In addition, it should be considered that pressure under the bandage is a dynamic parameter that varies with time, posture and activity, directly influencing the behavior of stiffness; therefore, continuous monitoring is essential to ensure the persistence of the therapeutic effect and to better understand the variations induced by movement [1,12].

In response to these limitations, current research has driven the development of new compression systems, incorporating various pressure sensors combined with wireless communication modules such as Bluetooth or inductive coupling technologies, designed to provide continuous monitoring if needed, along with real-time feedback, improving both patient adherence and accuracy in pressure dosing, if necessary [18,19,20].

Despite advances in measurement technologies, which now allow interface pressure and stiffness to be recorded at multiple points and under different conditions, there is still a substantial gap in the literature: a robust correlation between quantifiable biomechanical parameters (such as mean or final pressure and static or dynamic stiffness) and clearly defined, standardized clinical outcomes, such and edema reduction and wound area decrease in patients with VLU, has not yet been clearly established in the literature.

The complexity and variability inherent in the different compression systems (elastic, inelastic or multicomponent), together with the demonstrated influence of stiffness and dynamic pressure behavior on therapeutic efficacy, underline the need for comparative research that precisely links the biomechanics of the compression system with clinical response. This integration is essential to optimize treatment selection and maximize healing in VLUs.

## 2. Main Objective

To compare the clinical efficacy and biomechanical behavior of different compression systems in patients with venous leg ulcers, analyzing the relationship between interface pressure, static stiffness, and reduction in edema and lesion area.

## 3. Specific Objectives

To describe the relationship between the average pressure at the end of the study recorded by the three sensors and the reduction in leg contour in the different compression systems.

To compare the percentage of ulcer area reduction between the different compression products and to analyze their association with the final interface pressure.

To analyze the association between static stiffness in the three sensors and the reduction in the wound area, identifying differential patterns according to the compression system.

To explore how the combination of “static stiffness × final pressure” relates to the overall clinical efficacy of each system, identifying those bandages with the best performance.

## 4. Methodology

Study Design: A prospective, observational and multicenter study was carried out in four provinces of Andalusia, which included six Health Districts (DS) and/or Health Management Areas (AGS): DS Almería, DS Metropolitano de Granada, DS Jaén-Jaén Sur, AGS Norte de Jaén, AGS Serranía de Málaga and AGS Este de Málaga Axarquía. This research is part of a larger project where the clinical efficacy of the different types of venous compression systems has been measured by Advanced Practice Nurses in Complex Chronic Wounds (EPA-HCC) in Andalusia (Spain).

Study population: The target population consisted of people with active VLU s referred to the EPA-HCC during the study period, which ran from January 2024 to July 2025.

Sample: This is a conceptual sample since the entire population referred to the EPA-HCC during the study period, and who met the eligibility criteria until the sample size was completed.

Inclusion and exclusion criteria: People with VLU, whose ankle–brachial index (ABI) was between 0.9 and 1.3, with exudate and scarring that would allow two weekly cures, ambulant, and over 18 years of age, belonging to the same SD and/or AGS, were included. Patients with other types of wounds, with an inability to give informed consent, or in an end-of-life situation were excluded.

Sample size: For the calculation of the sample size and according to the main objective of the study and to achieve an accuracy of 5.00% in the estimation of a proportion using a bilateral 95.00% normal asymptotic confidence interval, assuming that the proportion of pressure loss of the systems at 96 h is 10.00%, It was necessary to include 139 patients in the study. Patients who did not complete the study period due to problems with the bandage (pain, etc.) were excluded from the analysis.

Sample selection method: A non-probabilistic sampling of an intentional or convenience type was used, consecutively incorporating all patients who met criteria during the collection period.

As an observational study reflecting real-world clinical practice, the choice of the multicomponent compression system was not randomized, nor was it targeted based on specific patient profiles or wound characteristics. Instead, the application of a specific brand depended entirely on its availability at each participating health center and the standard routine protocol of the treating Advanced Practice Nurse at that time. This convenience sampling approach accurately reflects standard outpatient care and prevents targeted selection bias by the researchers.

Variables analyzed:

1. Sociodemographic variables: Age; gender; weight; height; calculated BMI; and the care setting.
2. Initial description of the ulcer: Etiology; surface area measured by IMITO^®^ application previously calibrated with the markers of the application, both at the beginning and at the end of the study; location; affected limb; ABI; and initial and final RESVECH 2.0.
3. Data on the initial and final perimeters at the three basic points (B: supramalleolar; B1: Achilles tendon-gastrocnemius transition; and C: twin).
4. Compression materials used: Grouped by the systems studied: Circaid Juxtalite^®^ (medi GmbH & Co. KG, Bayreuth, Germany), CPK Compress; CPK Compress^®^ 2^®^ (CPK C2) (Farmaban S.A., Barcelona, Spain); Urgo K1^®^; Urgo K2^®^ (Urgo Medical, Chenôve, France); and Jobst Compri2^® ^(Essity AB, Gothenburg, Sweden). It is important to note that all the multicomponent compression systems included and evaluated in this study belong to the short-stretch (inelastic) category. Therefore, the comparative analysis focuses on the differences between various configurations within this specific typology.
5. Pressure at points B, B1 and C at the time of bandage placement according to the manufacturer’s instructions, and at 24, 48, 72 and 96 h, in resting conditions (recumbency, sitting, standing) and during activity (supine dorsiflexion and standing gait), with calculation of the dynamic stiffness index (DSI) and the static stiffness index (SSI); however, the results presented in this manuscript correspond to the 96 h measurements.
6. The dressings were performed before the initial measurement and after the 96-h measurement.

Data collection methods and instruments: Prior to the start of the study, the collaborating companies (Farmaban^® ^[Farmaban S.A., Sant Fruitós de Bages, Barcelona, Spain], Urgo^® ^[Urgo Medical, Chenôve, France], Medi^®^ [medi GmbH & Co. KG, Bayreuth, Germany] and Essity^® ^[Essity AB, Gothenburg, Sweden]) were asked to provide compression materials and specific training aimed at standardizing the technique and reducing biases. In addition, the company Feeltect^®^ was trained in the operation of the measuring device. All companies except Essity^®^ participated in the EPA-HCC training, accreditation and certification for the placement of their devices.

Although Essity^®^ did not provide training or materials, its system was included to maintain the independence of the study, which did not receive external funding. An external company supplied the 7.5 and 10.5 cm × 10 m tubular bandages used for the study and applied to all the systems evaluated. It was found that this tubular mesh did not alter the final compression results after bandages.

The pressure exerted by the compression devices at different times, on the different points, and in the different positions was monitored through the FeelTect^®^ Tight Alright^®^ system. This system consists of: a portable rechargeable transmitter, a sensor with disposable adhesive sleeves, and associated software installed on the EPA-HCC Ipads. Sensors in small, medium and long sizes made it possible to measure according to the patient’s size, at three points on the leg (B, B1 and C) to obtain a complete compression profile. They were placed on a tubular mesh (7.5 and 10.5 cm × 10 m), avoiding direct contact with the skin and thus possible injuries.

The procedure, once the cure was carried out, consisted of placing the tubular bandage (7.5 and 10.5 cm × 10 m) according to the perimeters of the patients’ legs, placing the sensors, applying the compression system that uses visual indicators of tension or stretch according to the manufacturer’s indications with the leg in a physiological position and recording the pressures in real time.

**Figure 1:**
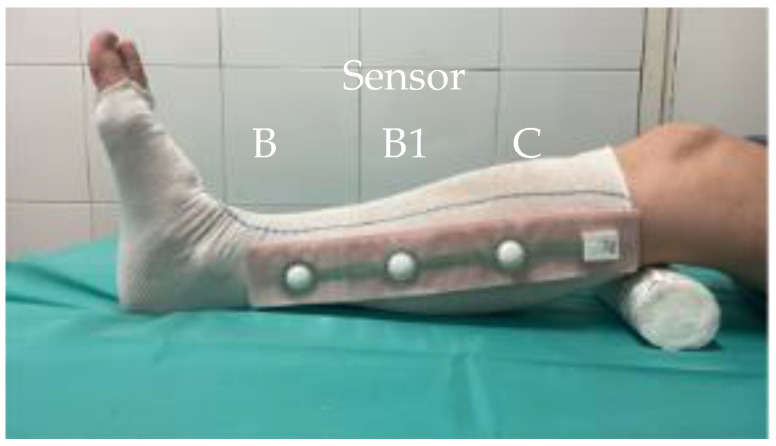
Placement of the sensors inserted in an adhesive sleeve between the tubular mesh and the compression system to avoid direct contact with the skin.

**Figure 2:**
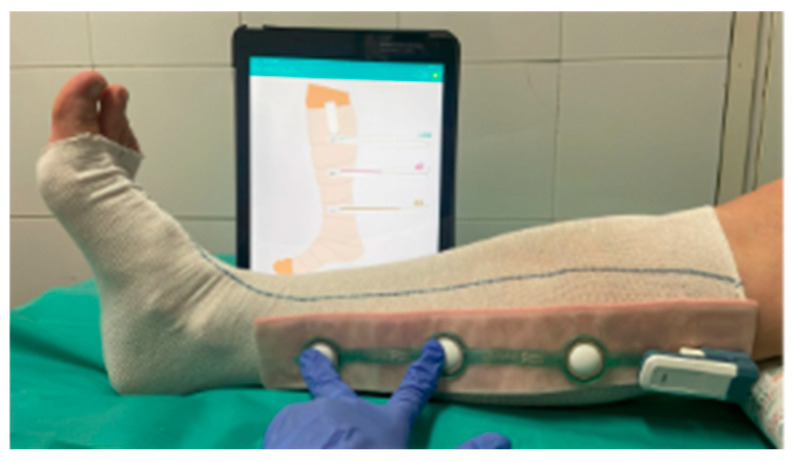
Checking sensors at B and B1 with an iPad before applying the compression systems.

A modified technique based on Fisher was used for a low-elasticity bandage with the CPK Compress system. The patient remained supine and had dorsiflexion of 30° after healing and placement of sensors. The procedure included placing the same tubular bandage (from the root of the fingers to the popliteal hollow), followed by a CPK Compress 8 cm × 7 m bandage using the herringbone technique and 50% stretching and overlapping. A second CPK Compress 10 cm × 7 m bandage was added, starting on the malleoli and ending under the tibial tuberosity, also in a spike and with 50% tension and overlap. The equipment was tested before each use, and the measurements were made at rest and during activity to assess the functional response of the patient, which can be seen in Figure 1 and Figure 2.

The device showed results in mmHg (values < 20 mmHg, such as “LOW”), being safe, and did not require removal of sensors due to adverse effects. It is classified as a Class I medical device, and complies with CE, FDA and UKCA regulations.

In addition, each EPA-HCC applied local care protocols following the acronym TIMERS (tissue, infection/inflammation, moisture, epithelialization, repair/regeneration and support), adapted to the healing phase at baseline. This allowed for individualized interventions, ensuring the effectiveness of the compression treatment and patient safety.

For the collection, an Ad hoc questionnaire was developed to record all the variables.

**Data analysis:** Descriptive statistics were used to summarize the clinical and biomechanical variables. Quantitative data are presented as means, standard deviations (SD), and confidence intervals 95%. To ensure an appropriate visual representation of data distribution and variability across the different multicomponent compression systems, box-and-whisker plots were generated, with the X-axes logically ordered according to the ascending values of the independent variables. To evaluate the strength and direction of the linear associations, Pearson’s correlation coefficient (R) was utilized. Furthermore, simple linear regression analyses were performed to comprehensively explore the individual relationships between clinical efficacy (percentage of wound area reduction as the continuous dependent variable) and each of the primary biomechanical parameters (final interface pressure, static stiffness index [SSI], and dynamic stiffness index [DSI] across the three sensor points as independent predictors). For all regression models, the coefficient of determination (R^2^) and the exact *p*-values were calculated. A *p*-value of <0.05 was considered statistically significant for all analyses. Statistical processing was conducted using SPSS 24.0 software.

**Ethical aspects:** The project (AP-0455-2023-C4-F2) was approved by the Coordinating Committee on Research Ethics in Almería on 9 November 2023, which confirmed that the study respected the ethical principles established in the 2013 Declaration of Helsinki for biomedical research with human subjects. The risk/benefit ratio was ensured, avoiding unnecessary inconvenience to patients, adapting data collection to their needs, preferences and search for their well-being. They were informed of the right not to answer any questions.

All participants received, prior to inclusion, a fact sheet about the study that was reinforced with verbal explanations before signing the informed consent. They were guaranteed the voluntary nature of their participation, without prejudice to receiving the best possible attention and treatment at all times, in addition to the confidentiality of the data, and they were offered the option of withdrawing at any time in accordance with Organic Law 3/2018 on the Protection of Personal Data and guarantees of digital rights. The rest of the ARCO rights were guaranteed.

To guarantee the confidentiality of the data provided by the participants, as well as their anonymity, the names of the participants were changed to codes.

## 5. Results

The analysis of the collected data provides a comprehensive overview of the performance of the compression systems evaluated, focusing on both their biomechanical behavior and their direct clinical impact. To offer a clear understanding of these interactions, the findings are presented by systematically linking the physical variables monitored—interface pressure and static stiffness—with key therapeutic outcomes, such as peripheral edema reduction and the percentage decrease in ulcer area. The following sections detail the decongestive effect across different anatomical points, followed by a comparative analysis of clinical efficacy between brands, aiming to discern whether healing rates are primarily driven by pressure magnitude or by the intrinsic technological design and stiffness of each system.

Table 1 shows the mean final pressure (on the fourth day) of the systems at the three points, together with their confidence interval and standard deviation.

As can be seen, a total of 140 patients have been included. The distribution of patients among the different systems was very homogeneous. All of them maintained mean pressures between 30 and 40 mmHg (including the 95% confidence interval) on the fourth day as an average among the three sensors.

**Graph 1:**
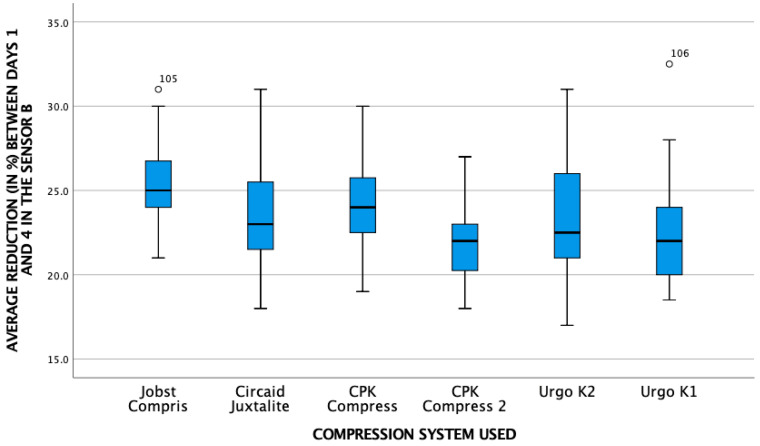
Relationship between average pressure and reduction in leg contour at point B.

**Graph 2:**
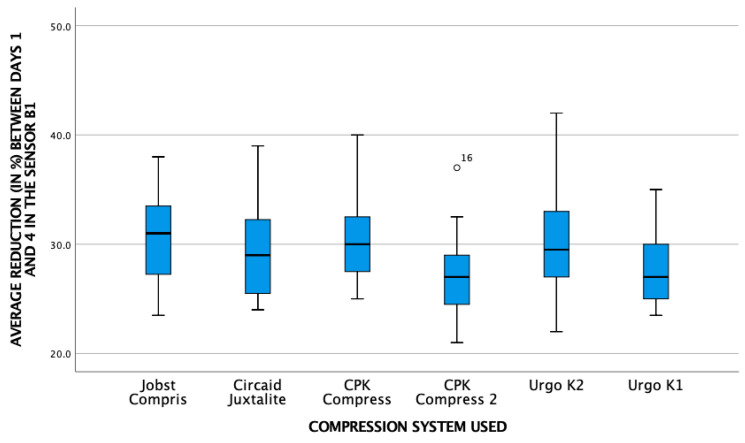
Relationship between average pressure and reduction in leg contour at point B1.

**Graph 3:**
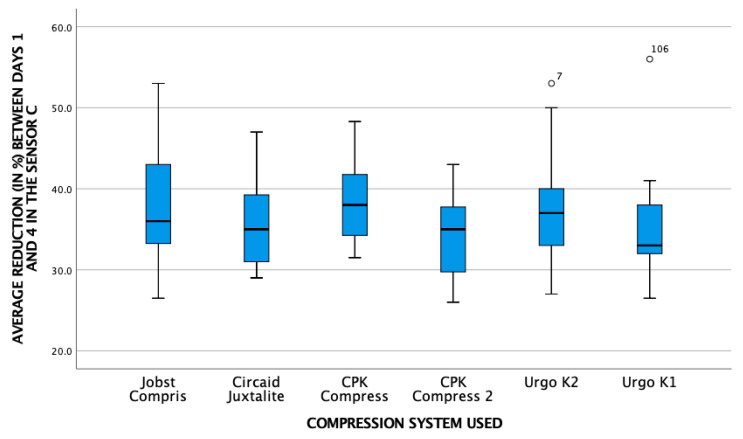
Relationship between mean pressure and reduction in leg contour at point C.

Graph 1, Graph 2 and Graph 3 show the relationship between the average pressure of the different pressure systems and the reduction in leg contour in the three sensors.

The graph illustrates an overall reduction in leg circumference. Visually, this decongestive effect exhibits a trend towards being more prominent at the B1 sensor point compared to the B sensor. This decongestive effect was highly significant across all measured points (*p* < 0.001).

Table 2 provides a more detailed analysis of the overall reduction in all systems in the supine position between day 0 (application of the bandage) and day 4 (day of the final analysis).

This confirms the findings of the previous graphs. The reduction in swelling was statistically more prominent at sensor point B1 compared to sensor B (mean reduction 19.4 vs. 17.3; *p* < 0.01). Furthermore, at the proximal point, the distribution of the data across the different brands displays considerable variability. Based on these graphical observations, there is no obvious visual pattern to suggest a direct proportional relationship between the final local pressure recorded and the magnitude of circumference reduction, pointing instead towards a multifactorial biomechanical behavior among the different multicomponent systems.

**Graph 4:**
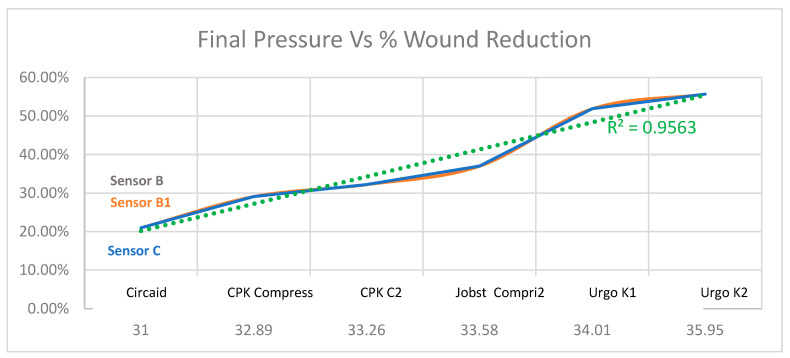
Relationship between final pressure and percentage reduction in wound size in B, B1 and C sensors.

Graph 4 shows the relationship between final pressure and percentage reduction in wound size.

Unlike the graph above, here we see huge differences in clinical efficacy between products. Urgo K2^®^ is the one that achieved the greatest wound reduction of 55.70%. Interestingly, it registered a pressure of 34.01, not being the highest, followed by CPK COMPRESS 2^®^, with a reduction of 51.90% and the highest pressure recorded in the group (35.95).

By contrast, Urgo K1^®^ had the worst result with only a 21.00% drawdown. It is interesting to compare these two products of the same brand, Urgo K1^®^ with a pressure of 33.35, 38.75 and 32.89 in sensors B, B1 and C, which achieves a cure of 21% and Urgo K2^®^ with much higher pressures in sensor B and B1 and more similar in sensor C achieves more than double the cures of 55.7%.

Systems such as CPK COMPRESS 2^®^ and Urgo K2^®^ achieved the highest wound reduction rates. Simple linear regression analysis, using the mean interface pressure values from all measurement points (detailed in Table 1) against the clinical success rate, confirms that there is no significant linear correlation (Pearson’s correlation coefficient r = 0.105; R^2^ = 0.011, *p* = 0.697).

This strongly suggests that the superior clinical efficacy of these specific brands is not strictly pressure-dependent, but rather driven by the multifactorial synergy of their multicomponent design and intrinsic stiffness.

**Graph 5:**
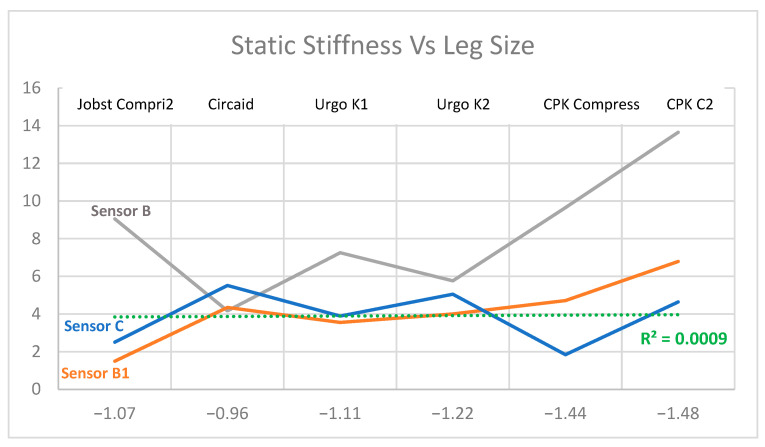
Relationship between static stiffness and wound area at point B, B1 and C.

That is why, in Graph 5, we compare static stiffness and wound area.

Sensor B shows that CPK^®^ products (Compress and COMPRESS 2) generate the “lowest” values (most negative, approx −1.2 to −1.3) on this sensor, in contrast to Circaid and Urgo, which maintain values closer to zero.

If we cross-referenced the data from the three sensors, we discovered something interesting about the use of the products: the nurses have unconsciously chosen the CPK and Josbt Compri 2^®^ products for the larger leg sizes, while Circaid^®^ and Urgo K2^®^ have been put on the smaller legs.

The “V” drop in CPK products shows that there is a huge drop in the rigidity of these products. Again, the low correlation shows that stiffness does not depend on leg size. The drop in the graph is not because the leg is large, but because CPK products were used in those large legs, which have different stiffness properties.

However, the C sensor displays different data than the B and B1 sensors. For Sensor C, the product that registers the lowest value (most negative, −1.72) is Urgo K2^®^, while in Sensor 1, Urgo K2^®^ had normal values. This indicates that the distribution of pressure/stiffness is not uniform along the leg. The Urgo K2^®^ bandage behaves very differently depending on where the sensor is placed (ankle vs. calf area).

**Graph 6:**
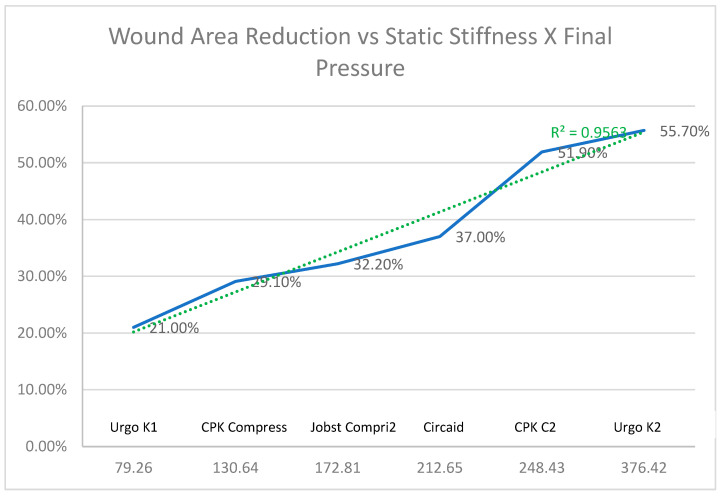
Relationship of the reduction in the wound area vs. static stiffness multiplied by the final pressure.

Finally, Graph 6 represents the relationship between the reduction in the wound area versus the static stiffness due to the final pressure.

As shown in Graph 6, there is a strong positive correlation (R2 = 0.9563) between the wound area reduction and the combined variable of static stiffness x final pressure. We can analyze the individual performance of each compression system. Urgo K2^®^ shows the best result with an area reduction of more than 55%, followed by CPK COMPRESS 2^®^ with a reduction of close to 52%. Circaid^®^ is with 37%, Jobst Compri2^®^ with 32%, and CPK Compress^®^ with around 29% show an average performance, while Urgo K1 shows the worst result in this group, with a reduction of close to 21%. The graph clearly demonstrates that success in reducing the wound area varies drastically depending on the brand and system used, and this variation is, in fact, strongly explained by the increase in the “Static Stiffness x Final Pressure”. Consequently, based on this strong mathematical correlation and clinical results, Urgo K2^®^ and CPK COMPRESS 2^®^ are shown as the most clinically effective options.

To comprehensively evaluate the biomechanical drivers of wound healing, multiple linear regression analyses were performed. The comprehensive results of these models are detailed in Table 3.

As shown, all models yielded extremely weak correlations and low explained variances, demonstrating no statistically significant linear association between the isolated biomechanical parameters (final interface pressure, static stiffness, and dynamic stiffness) and the reduction in ulcer area. From a clinical perspective, the absence of a direct linear correlation is a highly relevant finding. It quantitatively demonstrates that ulcer healing is not a simple linear function of applying progressively higher pressure or achieving higher numerical stiffness. Instead, it supports the premise that once a therapeutic biomechanical threshold is reached, successful wound closure is primarily driven by the holistic design of the multicomponent compression system (including material synergy, friction, and sustained wearability) rather than by isolated physical variables.

## 6. Discussion

This multicenter study addressed a persistent public health challenge: the effective management of VLU in the lower extremities, knowing that not all multicomponent compression systems are equivalent from a clinical or biomechanical point of view, and that interface pressure and stiffness provide complementary but not sufficient information alone to explain the healing of venous ulcers [21,22].

The findings indicate that some systems, such as Urgo K2^®^ and CPK COMPRESS 2^®^, achieve significantly higher wound area reductions than others, despite sharing similar pressure ranges, which underlines the determining role of the design and dynamic behavior of the bandage. Overall, the results support the hypothesis that pressure matters, but that the interaction between stiffness, spatial distribution of compression, and selection of the system according to the patient’s profile critically conditions the clinical outcome.

In relation to the interpretation of the results, all the systems evaluated achieved a reduction in leg circumference, more marked in the proximal sensors than in the distal sensors, suggesting a generalized decongestant effect of compression therapy regardless of the brand.

However, the absence of a clear linear correlation between the final pressure measured at each sensor and the magnitude of the edema reduction indicates that the mere “amount” of pressure does not fully explain the limb response, probably due to the influence of leg morphology, material distribution, and load pattern during ambulation [21,23,24,25].

When the reduction in wound area is analyzed, clinically relevant differences emerge: Urgo K2^®^ and CPK COMPRESS 2^®^ achieve reductions of more than 50%, while other systems remain in medium or low ranges, despite comparable pressures, which suggests that the intrinsic biomechanical properties and the mode of application modulate the effective transmission of pressure to the venous and tissue bed [21,26,27,28].

Graphs combining static stiffness and leg size show complex patterns, with a marked drop in stiffness in CPK products in larger leg circumferences and more stable values in systems such as Circaid^®^ or Urgo K2^®^ in smaller limbs. This behavior suggests that the interaction between the material and the diameter of the limb conditions the effective stiffness achieved, and that the nurses are already unconsciously adjusting the choice of the system to the size of the leg, which could be influencing the observed results.

Likewise, the fact that Urgo K2^®^ has very different stiffness values between the ankle and calf indicates that the longitudinal distribution of pressure is not homogeneous and that certain systems can generate more favorable compression profiles for venous hemodynamics in specific areas, which could explain part of its better clinical performance.

When comparing these findings with the literature, it is confirmed that effective compression improves the healing of venous ulcers and reduces edema, but also that the optimal magnitude of pressure and the role of stiffness remain topics of debate [5,14,15]. Previous studies have reported that ankle pressures around 40 mmHg and systems with high static stiffness are associated with better calf muscle pump function and a higher venous ejection fraction, although there is not always a direct correlation between these parameters and ulcer area reduction in routine clinical practice [5,14,15]. Our results are consistent with this evidence, but they also add an important nuance: the overall statistical correlation is weak, and the variability between brands suggests that factors such as layer composition, elasticity, and pressure stability are as relevant as the absolute value of the pressure or stiffness.

From the perspective of clinical relevance, the data reinforce that the choice of compression system cannot be based only on the “high” or “low” compression category, but must consider the actual performance of the dressing in terms of edema reduction and wound area. The fact that the same pressure range translates into such disparate results between Urgo K1^®^ and Urgo K2^®^ illustrates that the multicomponent design, the ability to maintain pressure over time, and the stiffness profile along the leg directly influence healing, so they should be incorporated into clinical decision algorithms [12,22,29,30].

In addition, the use of multipoint monitoring with Tight Alright^®^ and the estimation of static stiffness provides a relevant methodological contribution, by allowing to link for the first time, in a multicenter environment and with real patients, pressure and stiffness patterns with standardized results such as RESVECH 2.0, perimeters and wound area [20].

Among the unexpected findings, on the one hand, the weak overall correlation between final pressure and reduction in ulcer area stands out, and on the other, the worse performance of a system of the same brand (Urgo K1^®^) compared to its counterpart (Urgo K2^®^), despite apparently similar pressures, is equally significant. One possible explanation is that differences in elasticity, number of layers and stress stability condition the ability to maintain an effective gradient during movement, so that more rigid or better stabilized systems provide a superior hemodynamic effect without the need to excessively increase the resting pressure.

In addition, the drop in stiffness observed in the CPK Compress and Compress 2 products in larger legs could reflect limitations of the material to maintain stiffness when stretched above certain thresholds, which would reduce its effectiveness in limbs with a larger perimeter, despite still registering reasonable pressures at specific points.

It is noteworthy that the two bandage models with the lowest clinical efficacy in terms of their biomechanical behavior are the CPK Compress^®^ and the UrgoK1^®^, and they have the fact that they are cohesive bandages in common, unlike others (CPK COMPRESS 2^®^ and UrgoK2^®^) that are not. This raises another possible dependent variable that would require further studies for confirmation, and that is whether the cohesiveness of the bandages influences the final rigidity of the bandage, thus affecting static rigidity and clinical efficacy, as it seems to be.

The study has several strengths that increase the validity of its conclusions, including the prospective and multicenter design in several Andalusian health districts and the use of a continuous and multipoint pressure monitoring device that provides objective measures of pressure and stiffness in real conditions of clinical practice.

The participation of EPA-HCC guarantees a high degree of standardization in the application of the systems and in the local management of the ulcer, supported by TIMERS protocols, which reduces the variability attributable to the technique [31,32]. However, there are also important limitations: sampling is convenient, the assignment of the compression system was not random, and the duration of follow-up is limited to 96 h for biomechanical parameters, which makes it impossible to capture the entire healing process and its temporal relationship with the evolution of pressure [20,24].

In addition, the sample size was calculated based on pressure loss at 96 h and not specifically to detect clinical differences in ulcer area reduction between systems. Finally, the stiffness measurement is based on a limited number of points (B, B1 and C) and does not capture the three-dimensional complexity of the pressure distribution, so local variations with impact could go unnoticed.

Finally, although the stiffness measurement is based on a limited number of points (B, B1 and C) and does not capture the three-dimensional complexity of the pressure distribution, which could leave local variations with clinical impact unnoticed, most devices for measuring pressure in therapeutic compression use a single sensor, which represents an even greater limitation [33,34].

In terms of practical implications, the results support that compression system selection protocols for venous ulcers incorporate not only the target pressure level, but also data on the comparative clinical performance of each system and its stiffness behavior in different leg morphologies [22,35].

Systems that have shown better results in reducing the ulcer area, such as Urgo K2 and CPK COMPRESS 2, could be prioritized in patients with VLU of greater complexity or in contexts where pressure monitoring is available, always considering contraindications and patient preferences.

In terms of future lines of research, it would be desirable to develop comparative clinical trials with random assignment of the different compression systems, with longer follow-ups that allow a robust relationship between pressure and stiffness patterns in the first 96 h and complete healing and medium-term recurrences.

Analyses that integrate variables of cost, comfort and adherence perceived by patients are also a priority in order to move towards decision models that balance clinical efficacy, tolerability and economic efficiency in the context of public health systems.

In summary, this study shows that, in the management of venous leg ulcers, the effectiveness of compression depends both on how much is compressed and on how and with what system it is performed, and that there are relevant differences between the bandages available in terms of edema reduction and area of injury.

The integration of objective pressure and stiffness measurements into advanced nursing practice allows for progress towards a more rational, data-driven, patient-tailored understanding and provides useful evidence to optimize system selection and improve healing outcomes in actual clinical practice.

Crucially, the application of multivariable regression models in our study provided a more realistic understanding of the biomechanical drivers of wound healing. By analyzing multiple independent predictors simultaneously, we demonstrated that isolated physical parameters—such as interface pressure peaks or static stiffness alone—explain only a minor fraction of the variance in ulcer reduction. This multivariable approach confirms that therapeutic success cannot be attributed to a single variable. Rather, it is the result of a complex multifactorial interplay, emphasizing the importance of the integral multicomponent design, material synergy, and sustained wearability of the compression systems.

## 7. Conclusions

In conclusion, this multicenter study shows that the clinical efficacy of multicomponent compression systems in VLU depends not only on achieving a certain pressure level, but also on the biomechanical behavior of each system, including stiffness and the longitudinal distribution of pressure. Systems such as Urgo K2^®^ and CPK COMPRESS 2^®^ achieved greater reductions in wound area despite similar pressure ranges, highlighting the importance of design and pressure stability over time. Multipoint monitoring of interface pressure and stiffness with devices such as Tight Alright^®^ provides valuable information to guide the selection of compression systems and supports the incorporation of biomechanical parameters into clinical decision-making.

## Figures and Tables

**Table 1:** Mean of final pressure (on the fourth day) of the different systems used at the three points (B, B1 and C).

System	N	Average	CI 95%		SD
Circaid Juxtalite	23	38.15	36.19	40.11	4.54
CPK Compress	23	33.46	33.30	33.62	0.37
CPK Compress 2	23	33.40	32.32	34.49	2.51
Jobst Buy2	23	33.49	32.43	34.55	2.45
Urgo K1	23	35.00	33.59	36.41	3.26
Urgo K2	25	42.85	39.60	46.10	7.87

**Table 2:** Analysis of the overall reduction in all systems used in the supine position between day 0 (application of the bandage) and day 4 (day of the final analysis).

		Mean	Standard Deviation	95% Confidence Interval for the Difference		*p*-Values
				Lower	Upper	
Pair 1 (sensor B, days 0 and 4)	Data analyzed for sensor B on days 0 and 4	19.427	16.419	16.421	22.434	<0.0001
Pair 2 (sensor B1, days 0 and 4)	Data analyzed for sensor B1 on days 0 and 4	17.397	14.570	14.774	20.019	<0.0001
Pair 3 (sensor C, days 0 and 4)	Data analyzed for sensor C on days 0 and 4	15.718	11.522	13.541	17.896	<0.0001

**Table 3:** Multiple linear regression analysis to evaluate the biomechanical drivers of wound healing.

Regression Models (Independent Predictors)	Dependent Variable	R^2^	*p*-Value	95% Confidence Interval (CI)
Model 1: Final Interface Pressure (Sensors 1, 2, 3)	Ulcer Area Reduction (%)	0.011	0.697	S1: [−0.58, 1.51] S2: [−0.83, 0.67] S3: [−0.91, 0.50]
Model 2: Static Stiffness Index—SSI (Sensors 1, 2, 3)	Ulcer Area Reduction (%)	0.022	0.135	S1: [0.25, 0.35] S2: [−0.28, 0.16] S3: [−0.33, 0.13]
Model 3: Dynamic Stiffness Index—DSI (Sensors 1, 2, 3)	Ulcer Area Reduction (%)	0.086	0.871	S1: [−0.23, 0.24] S2: [−0.23, 0.53] S3: [−0.45, 0.32]

CI: Confidence interval calculated for the regression coefficients. A *p*-value < 0.05 indicates statistical significance.

## Data Availability

The original contributions presented in this study are included in the article. Further inquiries can be directed to the corresponding author.
